# First-in-human pilot study of snapshot multispectral endoscopy for delineation of pituitary adenoma

**DOI:** 10.1117/1.JBO.30.5.056002

**Published:** 2025-05-07

**Authors:** Dale J. Waterhouse, Daniele Borsetto, Thomas Santarius, James R. Tysome, Sarah E. Bohndiek

**Affiliations:** a University of Cambridge, Department of Physics, Cambridge, United Kingdom; b University of Cambridge, Cancer Research UK Cambridge Institute, Cambridge, United Kingdom; c Addenbrooke’s Hospital, Cambridge University Hospitals NHS Foundation Trust, ENT Department, Cambridge, United Kingdom; d Addenbrooke’s Hospital, Cambridge University Hospitals NHS Foundation Trust, Neurosurgery Department, Cambridge, United Kingdom

**Keywords:** multispectral imaging, endoscopy, pituitary adenoma, transsphenoidal surgery, spectral angle analysis

## Abstract

**Significance:**

The definitive treatment for pituitary adenoma is transsphenoidal surgical resection. Conventional white light imaging shows limited contrast between the adenoma and the pituitary gland, and only the tissue surface is visualized, leaving a pressing unmet need for improved intraoperative adenoma delineation to preserve pituitary function during surgery.

**Aim:**

To evaluate the potential of multispectral imaging to enhance visualization of adenoma during transsphenoidal resection.

**Approach:**

A multispectral camera based on a spectrally resolved detector array was coupled to a standard 4-mm rigid endoscope for *in vivo* imaging, such that the camera head could easily be switched with the standard of care camera head during surgery.

**Results:**

The multispectral imaging (MSI) endoscope was deployed during transsphenoidal surgery, and usable data were obtained from 12 patients. MSI was able to distinguish between an adenoma and a healthy pituitary based on the spectral angle with the reference spectrum of blood.

**Conclusions:**

The MSI endoscope holds the potential to differentiate adenoma tissue and healthy pituitary. With further development, MSI endoscopy could enable real-time label-free delineation of tumors during surgery, based on quantitative thresholds, which should contribute to improving the completeness of resection, while helping to preserve the pituitary gland, preventing serious life-changing complications.

## 1 Introduction

The pituitary gland is a pea-sized organ situated behind the ridge of the nose, attached to the base of the brain by a thin stalk. It is a key component of the endocrine system responsible for hormonal control of other glands, as well as many aspects of normal functioning including growth and blood pressure. Pituitary adenoma, a benign tumor, is the most common disease associated with the pituitary gland, with a prevalence of ∼17% in the U.S. population, although the majority cause no symptoms.^1^ Pituitary adenomas are diverse and may be functional or non-functional:^1^ functional adenomas usually present with clinical symptoms specific to increased hormone secretion, whereas non-functional adenomas present due to their mass, resulting in visual loss from compression of the optic chiasm, or as incidental findings on imaging for other indications.^2^

The definitive treatment for pituitary adenoma is transsphenoidal surgical resection.^3^ A surgeon inserts rigid endoscopic devices through the nostrils and to the back of the nasal cavity where small openings are made in three bones (the nasal septum, sphenoid sinus, and the sella) to reach the pituitary gland. The surgeon then removes the adenoma guided by white light imaging followed by closing the sella. Normal pituitary glands must be preserved to minimize loss of function, but contrast for pituitary adenoma under white light imaging is poor. Poor contrast impacts cure rates: for acromegaly-related adenomas, the cure rate is reported between 46% and 79%,^4^ whereas remission for Cushing’s disease-related adenomas is reported at 89%.^5^ For those with recurrent adenomas due to incomplete resection, repeated operation can be challenging due to scar formation^6^^,^^7^ and anatomical distortion,^8^ impairing resection and increasing risk of complications, such as loss of normal pituitary function and carotid artery rupture. Transsphenoidal surgery therefore requires a delicate balance between maximizing completeness of resection and preserving endocrine function from the normal pituitary.

Currently, surgeons rely on pre-operative magnetic resonance imaging (MRI) to locate the adenoma in relation to the normal pituitary gland. Post-operative MRI can then be used to assess the extent of resection; however, due to potential artifacts from hematoma or surgical packing, post-operative MRI often takes place months after the surgery.^9^ Intra-operative MRI (iMRI) can also be used, offering immediate feedback to surgeons following resection, but it is expensive and prolongs surgery.^9^ Moreover, image artifacts can lead to false positives, so iMRI is not recommended by the Congress of Neurological Surgeons.^3^

Intraoperative optical imaging technologies have been explored to provide real-time feedback in transsphenoidal surgery, including narrow-band imaging (NBI)^10^ (up to 4K ultra-high definition),^11^ indocyanine green (ICG) fluorescence imaging,^12^^,^^13^ and optical molecular imaging with a fluorescent folate analog.^14^ NBI^15^ uses two narrow illumination bands aligned with absorption peaks of hemoglobin in red blood cells (415±10 and 540±10  nm) to enhance contrast for vasculature. On standard NBI, the pituitary gland appears as an arabesque pattern due to the rich vasculature, whereas the adenoma has no particular pattern of enhancement,^10^ with far lower vascular density compared with the healthy anterior pituitary.^16^^,^^17^ Nonetheless, NBI still requires subjective interpretation and identification of margins can be difficult.

ICG is an intravenously administered fluorescent dye that binds to albumin, enabling vascular perfusion imaging and exploiting the aforementioned difference in pituitary and adenoma vascularity for contrast. ICG has a short half-life, an acceptable safety profile, and fluoresces in the near-infrared (NIR) allowing good depth penetration (up to a few millimeters),^12^^,^^13^ but procedural timing is vital to maximize contrast, given the intravenous nature of the administration.^18^ Pilot studies have found differences in ICG uptake in the pituitary gland compared with the adenoma,^13^ although the nature of the contrast appears to vary with the clinical symptoms of the patient^13^ leading to a lack of specificity.^12^ Furthermore, although NIR fluorescence endoscopes for ICG imaging are commercially available, they are currently larger in diameter than standard endoscopes and do not provide side-facing or tip-washing capability.^18^^,^^19^

To tackle the limited specificity of ICG, targeted intraoperative fluorescence imaging of folate receptors could be adopted, which has previously proven feasible and beneficial in ovarian cancer.^20^^,^^21^ Folate receptor alpha (FRα) overexpression has been reported in non-functioning pituitary adenomas.^22^^–^^24^ Using OTL38, a fluorescent folate analog, and a commercially available NIR fluorescence endoscope improved the true-positive and true-negative rates for detecting pituitary adenoma in margin specimens from 80% and 89% using white light alone to 86% and 89%, respectively, but many adenomas did not overexpress FRα (no functioning adenomas and half of the non-functioning adenomas overexpressed FRα), which remains a significant limitation.^14^

Considering the limitations of iMRI and optical surgical imaging tested to date, it is unsurprising that there has been no significant improvement in surgical outcomes over the last 30 years.^25^ Yet, the differences in vascularity between healthy pituitary gland and adenoma appear to be a promising route to achieving a contrast enhancement. Building on NBI, multispectral imaging (MSI) has the potential to use spatially resolved spectral data to classify disease based on endogenous contrast,^26^^,^^27^ overcoming the limitations of qualitative interpretation for NBI. NBI is a simple example of MSI, sampling just two colors. When light travels through tissue, it is absorbed by endogenous chromophores, such as hemoglobin (as exploited in NBI), and scattered by endogenous structures, such as cell nuclei.^28^ Disease-related structural and biochemical changes in tissue can alter the distribution and abundance of these absorbers and scatterers, resulting in subtle wavelength-dependent changes in reflected light, which can be measured by multispectral imaging methods that capture spatially resolved (x,y) and spectral (wavelength, λ) information.^26^^,^^27^ MSI has already shown promise for identifying tumors in a range of different organs but has yet to be examined for pituitary adenoma.^26^^,^^27^^,^^29^

Here, we evaluated the potential of MSI for high-contrast delineation of the pituitary gland and adenoma during transsphenoidal resection of the pituitary adenoma. A custom snapshot multispectral endoscope based on a spectrally resolved detector array (SRDA) was developed and applied in a first-in-human pilot clinical study to acquire *in vivo* MSI data from pituitary tissue and adenoma. The results demonstrate the potential for MSI to provide contrast between adenoma and healthy pituitary gland.

## 2 Methods

### 2.1 Endoscope Design

**Fig. 1:**
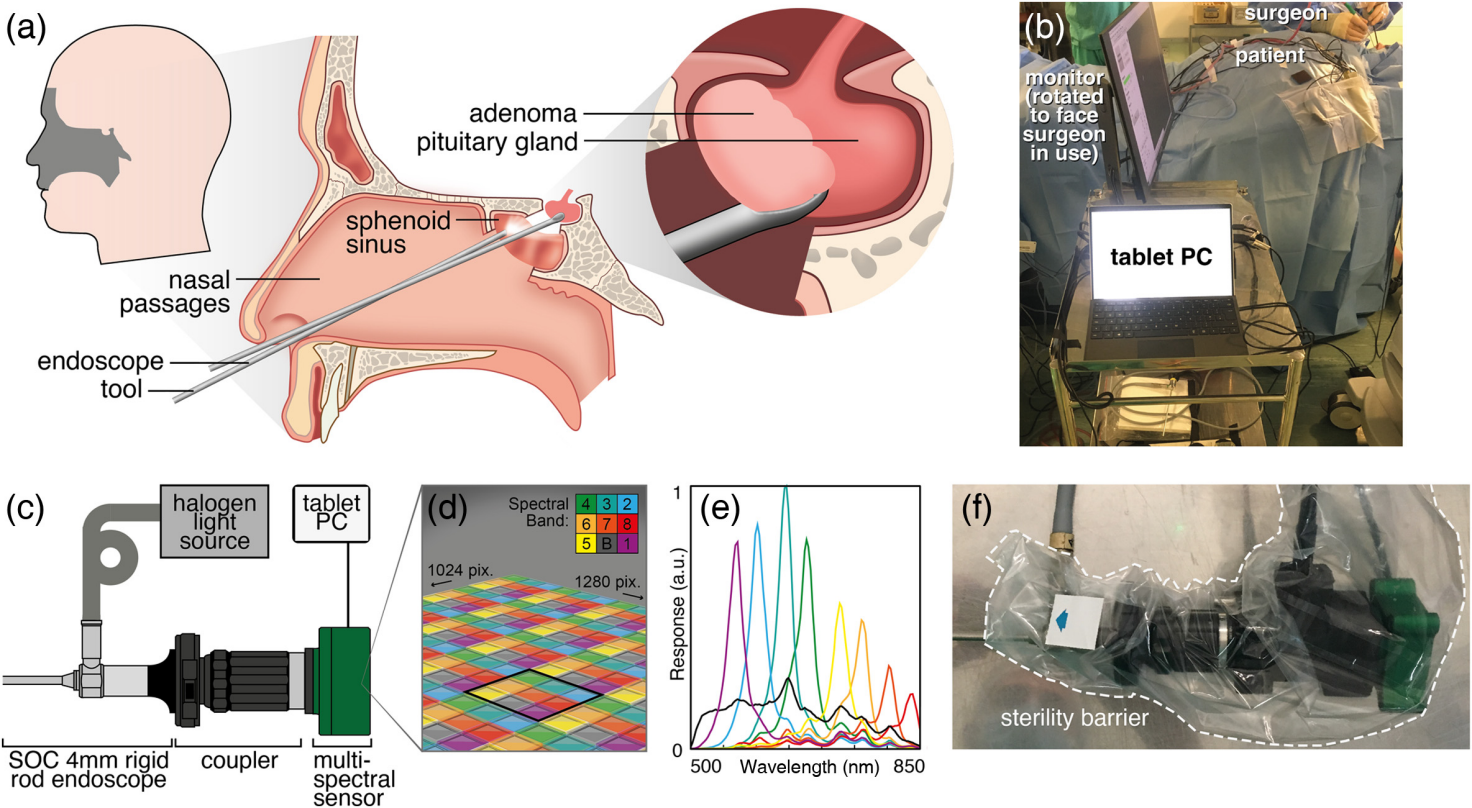
MSI endoscope design. (a) Schematic of transsphenoidal endoscopic resection of pituitary adenoma. (b) MSI endoscope was designed and constructed for use in a first-in-human pilot study, placed adjacent to the surgeon in the operating room. (c) MSI endoscope employs an SRDA with nine spectral filters (d) and (e) coupled to a standard 4-mm rigid endoscope for *in vivo* imaging. The MSI endoscope camera head can easily be switched with the SOC camera head. (f) SRDA camera head is covered by a sterile drape.

In transsphenoidal endoscopy, the back-end optics are clipped directly onto the rigid endoscope and handheld by the surgeon, so they must be compact and robust. We thus designed an MSI endoscope based around a compact SRDA [Figs. 1(a)–1(c)].^30^ Briefly, the system consists of a 4-mm 0° endoscope (Hopkins II, 7230AA, Karl Storz, Tuttlingen, Germany) coupled to an SRDA (CMS-V, SILIOS, Peynier, France) using a zoom coupler (18 to 35-mm zoom coupler, RVA Synergies, Gloucester, United Kingdom). The SRDA consists of nine spectral filters [Figs. 1(d) and 1(e)]; eight narrow bands; average full width at half maximum (FWHM) 30 nm; center wavelengths 553, 587, 629, 665, 714, 749, 791, and 829 nm; 1 broadband; and 500 to 850 nm, deposited as a 3×3 super-pixel across a CMOS sensor (NIR Ruby sensor, UI1242LE-NIR, IDS, square pixel size 5.3  μm). Illumination was provided by a broadband halogen light source (OSL2BIR, Thorlabs, Bergkirchen, Germany) with an extended illumination range into the near-infrared to cover the longer wavelength response of the SRDA. Illumination was coupled to the endoscope using a standard of care (SOC) fiber optic light guide.

For technical characterization, images were captured in uEye Cockpit (IDS, Germany) and saved as 8-bit BMP files. For clinical studies, the MSI endoscope settings were controlled using an interface developed in LabVIEW (National Instruments) running on a tablet (Surface Pro, Microsoft). For real-time surgical navigation, three of the nine spectral bands were displayed as a false-red-green-blue (RGB) colour image at up to 15 fps, whereas the raw mosaicked nine-band multispectral images were saved in the background as either 8-bit BMP files (to enable convenient review of the images) or binary files (to enable fast acquisition). Data analysis was carried out using MATLAB^®^ 2022a (MathWorks, Natick, Massachusetts, United States).

The MSI endoscope device architecture ensured that no modification was made to any part of the endoscope intended to be in direct patient contact, passing local clinical engineering approvals. The back-end optics were easily clipped on and off the rigid endoscope using a slider, ensuring minimal interruption to standard clinical workflow. As the back-end optics sit adjacent to the patient, they were covered with a plastic drape during surgery, as is standard protocol for rigid endoscopy cameras [Fig. 1(f)].

### 2.2 Technical Characterization

To determine the limiting resolution of the SRDA-based rigid MSI endoscope, images of a 1951 US Air Force (USAF) resolution test target (#53-714, Edmund Optics, Barrington, New Jersey, United States) were captured at six working distances (WDs) (2.5 to 15.0 mm) using external illumination from the broadband halogen light source (OSL2B2, Thorlabs, Bergkirchen, Germany) to reduce specular reflections. The raw images were demosaicked, and the broadband (500 to 850 nm) channel images were analyzed in grayscale.

To measure the field of view (FOV), images of a 1-mm checkerboard printed on white paper were captured at 17 WD, 4 to 20 mm (as measured using a translation stage, the distal tip of the endoscope initially in contact with the target, i.e., WD=0  mm, and then moved away using the screw gauge, error ±0.1  mm). The resulting images are expected to show barrel distortion defined by

$$r_{u}=Ar_{d}(1+kr_{d}^{2}),$$ (1)

where $r_{u}$ is the radial distance from the center of the ground truth image to a given vertex i in mm, $r_{d}$ is the radial distance from the center of the distorted image to the same vertex i in pixels, k is a constant that describes the magnitude of the distortion, and A is a constant used to convert among units of pixels and mm. For each of the images acquired, the position of the checkerboard vertices was identified using the built-in MATLAB function “detectCheckerboardPoints.” For each of these points, the radial distance to the center of the image, $r_{d}$ (in pixels), and the true distance to the center of the image, $r_{u}$ (in mm), which is known from the checkerboard pattern, were found.

### 2.3 Study Population and Design

This prospective pilot cohort study was carried out at Addenbrooke’s Hospital, Cambridge University Hospitals NHS Foundation Trust, Cambridge, United Kingdom. Eligible patients were adults (at least 18 years old) with planned surgery for pituitary adenoma. Patients with a significant illness or medical condition that made them unsuitable for endoscopic pituitary surgery were excluded. The trial was reviewed by the Health Research Authority and Health and Care Research Wales and was approved in August 2018 (18/EE/0165).

The Multispectral imaging of Adenoma during Pituitary Surgery trial was designed to address one primary objective and one secondary objective: (i) to evaluate the appearance of healthy pituitary tissue and pituitary adenoma at multispectral endoscopy to build a library of useful distinguishing characteristics because these are currently unknown and not easily investigated using *ex vivo* tissue and (ii) to assess the feasibility of imaging with the novel multispectral endoscope in endoscopic transsphenoidal pituitary surgery.

### 2.4 Surgical Procedure

**Fig. 2:**
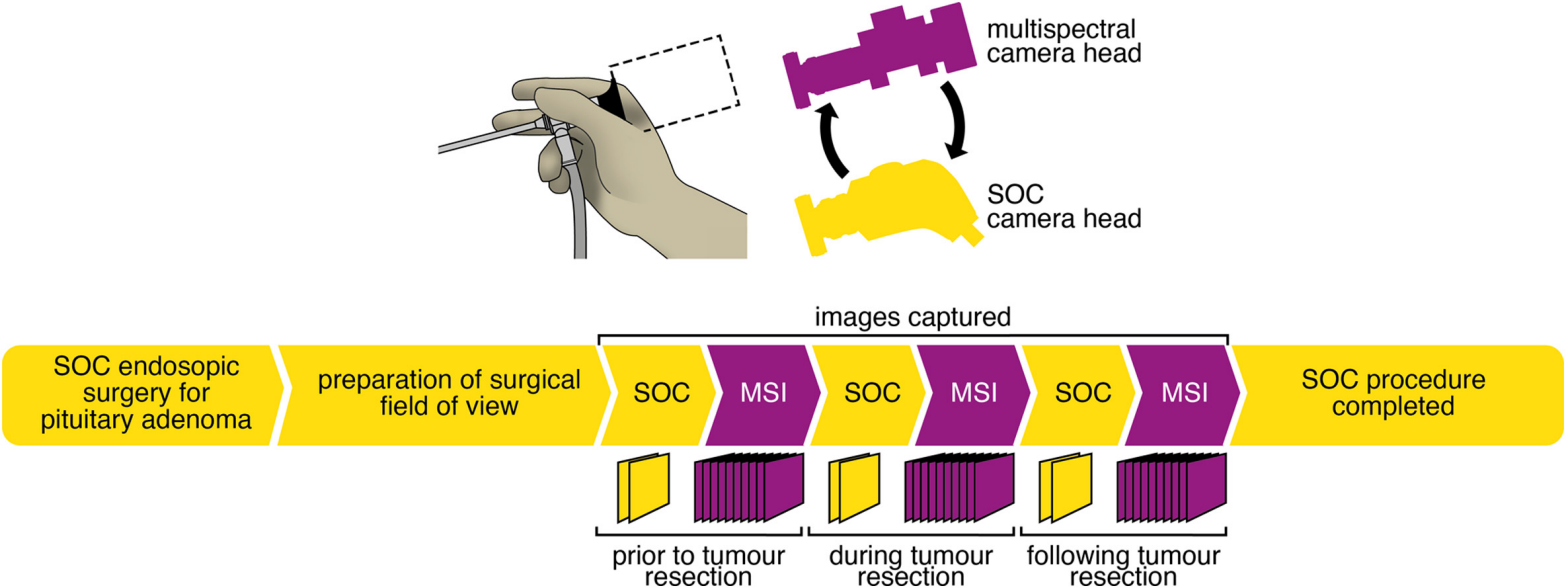
Schematic representation of the clinical trial protocol. Where possible, multispectral images were captured at three stages of surgery: (i) prior to tumor resection; (ii) during tumor resection, when the tumor was actively being removed but before complete resection was achieved; and (iii) following tumor resection, when resection was complete. SOC, standard of care; MSI, multispectral imaging.

Potential study participants were approached in the clinic after they had agreed to pituitary surgery for their pituitary macroadenomas. The study was explained, and patient information was given with time for reflection before consent was taken to enter the study. The surgical procedure was not modified in terms of patient preparation. Pituitary surgery proceeded with endoscopic resection of the pituitary adenoma using a standard two-surgeon four-handed technique. At three stages during tumor resection: (a) before the pituitary was seen, (b) when the pituitary gland was thought to be seen and, (c) when the tumor resection was finished, the surgeon captured a still SOC image with the standard white light endoscope. Next, the standard camera head was removed from the proximal end of the endoscope and replaced with the multispectral camera head to capture multispectral images of the same field of view (Fig. 2). Images were captured under illumination from the broadband halogen light source by switching the distal tip of the light guide among the light source output ports. The mean exposure time for multispectral image acquisition was 170±70  ms.

### 2.5 Delineation of Adenoma and Pituitary Regions in Multispectral Images

To enable the matching of multispectral image data with tissue types, the SOC images were annotated by the surgeon with regions of interest (ROIs) to delineate the tissues of interest (tumor and healthy pituitary) as well as other landmarks (e.g., dura of the sella). The multispectral images were processed to create a pseudo-RGB video. Pseudo-RGB images were generated by assigning the spectral bands with center wavelengths 629, 587, and 553 nm to R, G, and B channels, respectively, and performing a white balance correction to compensate for the lack of true blue light representation. Naturally, these images do not provide an equivalent white balance to the SOC image due to the red shift of the wavelengths used to compose the pseudo-RGB. The resulting video was carefully inspected to extract frames where the field of view matched that seen in the SOC images, allowing the annotations delineating the tissues of interest (tumor tissue and pituitary tissue) to be manually transferred from the SOC images to the multispectral images. During analysis, ROIs from regions where blood was visibly pooled were also drawn.

### 2.6 Image Processing

Raw images were checked for saturation (pixel values >245), dark subtracted, black level corrected, and demosaicked according to standard methods previously described^30^ to separate the nine spectral bands, creating an image cube. In the image cube, low signal pixels were removed (maximum<20 or minimum<0) as they are more likely to be affected by noise. Finally, each per-pixel spectrum was normalized to the area under curve (AUC) = 1 across the eight narrow bands. The mean spectrum per ROI was calculated by taking the mean over the remaining pixels within the ROI. The error was calculated as the standard deviation over the remaining pixels. ROIs with fewer than 10 remaining pixels were excluded as these were considered noise. The final spectrum for each ROI was normalized to AUC = 1 across the eight narrow bands.

### 2.7 Spectral Angle Calculation

To compare spectra, spectral angle calculations were performed. Spectral angle analysis calculates the n-dimensional angle between a target spectrum and a reference spectrum. The spectral angle between a spectrum $\overset{⇀}{s}$ and a reference spectrum $\overset{⇀}{r}$ is calculated as

$$\theta_{s-r}=\cos^{-1}(\frac{\overset{⇀}{s}\cdot \overset{⇀}{r}}{\left\|\overset{⇀}{s}\right\|\cdot \|\overset{⇀}{r}\|}),$$ (2)

where $\overset{⇀}{s}$ is the nine-band spectrum, $\vec{r}$ is a nine-band reference spectrum, · is the dot product, and $\left\|\overset{⇀}{r}\right\|$ represents the two-norm of the vector $\vec{r}$. The spectral angles compared the mean spectrum from each ROI with four reference spectra: the per-patient mean blood spectrum (to eliminate sources of inter-patient variation), the trial-wide mean adenoma spectrum, the trial-wide mean pituitary spectrum, and the trial-wide mean blood spectrum. Spectral angle calculation thus provides four spectral angles per ROI: θ-per-patient-blood, θ-adenoma, θ-pituitary, and θ-blood.

### 2.8 Statistical Analysis

Due to the exploratory nature of this study and the absence of a previously acquired dataset, a formal calculation of sample size was not possible. Our relatively small dataset contains both paired and unpaired data, so a partially paired t test (not assuming equal variances^31^) was used to assess pairwise differences among tissue classes.

## 3 Results

### 3.1 Technical Characterization Showed Suitable Imaging Performance for Surgical Testing

**Fig. 3:**
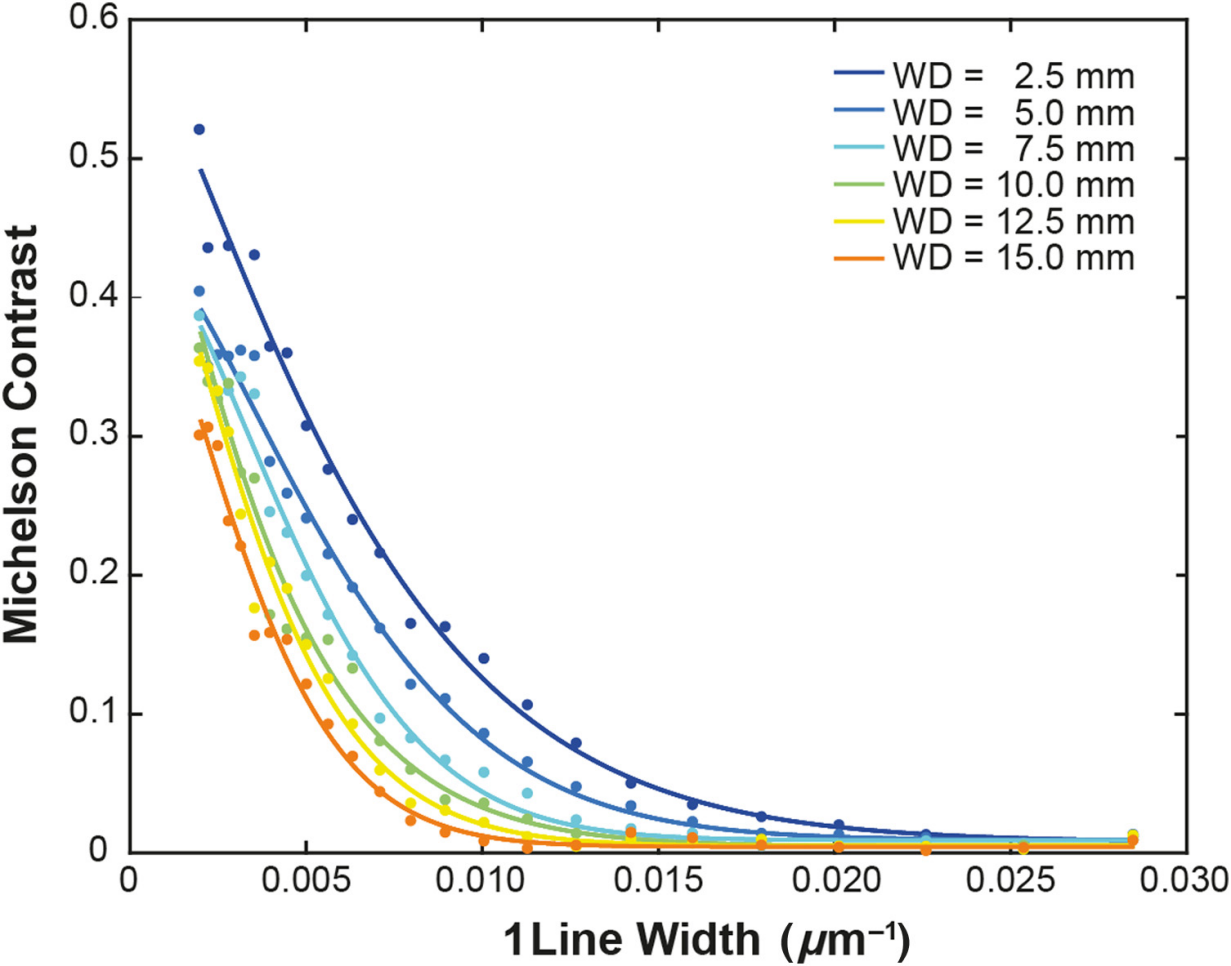
Resolution characterization using a USAF chart at six different working distances. The resolution was determined as the point where an exponential fit drops below 5% Michelson contrast. $R^{2}=0.9913$, 0.9909, 0.9896, 0.9785, 0.9862, and 0.9886 for working distances of 2.5, 5.0, 7.5, 10.0, 12.5, and 15 mm, respectively.

The presence of nine spectral bands on the SRDA meant that after demosiacking, the multispectral images had a limited resolution of (341×426  pixels) compared with SOC camera heads (up to ∼4000×2000  pixel 4K video). The Michelson contrast

$$C=(I_{\max}-I_{\min})/(I_{\max}+I_{\min}),$$ (3)

where $I_{\max}$ is the maximum intensity, and $I_{\min}$ is the minimum intensity, was calculated for each element of the USAF target and the results plotted against the reciprocal of the line width of the element (Fig. 3). A contrast threshold of 1% has previously been suggested,^32^ but a slightly higher threshold of 5% was chosen in this case to avoid effects arising from noise at very low contrast. By finding the intersect of this threshold with exponential fits applied to the data at each working distance, the resolution of the endoscope was determined to be in an acceptable range, with values of 68±7, 83±7, 103±9, 110±10, 130±10, and 150±10  μm, at working distances of 2.5, 5.0, 7.5, 10.0, 12.5, and 15 mm, respectively.

**Fig. 4:**
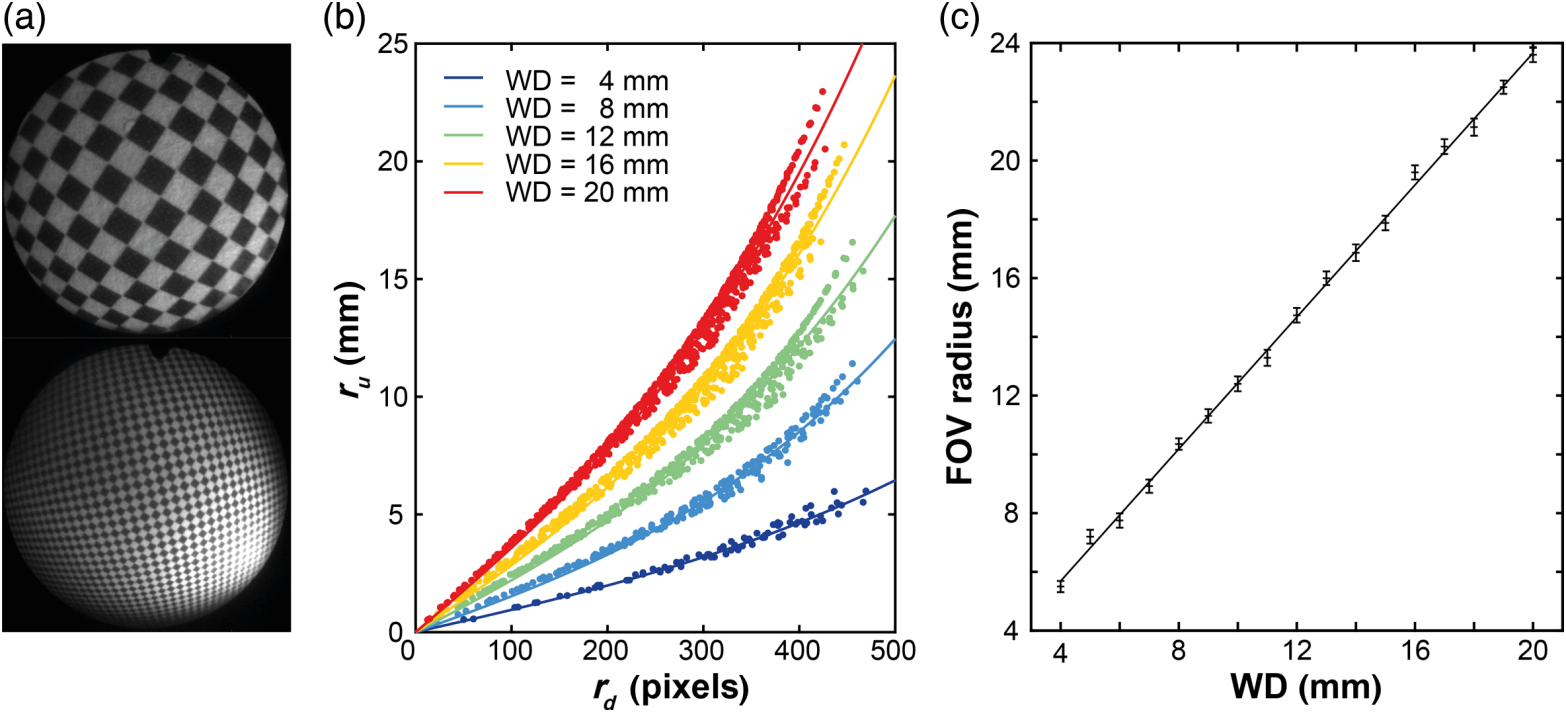
FOV of the MSI endoscope characterized using a checkerboard pattern. (a) Images of 1-mm checkerboard at WD = 4 and 20 mm show barrel distortion. (b) Data extracted from vertices of the checkerboard and fit to Eq. (1) for WDs of 4, 8, 12, 16, and 20 mm ($R^{2}=0.9799$ to 0.9908). The fit was used to extract the constant A and the distortion parameter k. The values of A and k can be used with Eq. (1) to determine the FOV radius ($=r_{u}$) based on the radius of the images in pixels ($=r_{d}$). (c) Determined FOV for 17 WDs ($R^{2}=0.9986$). Error bars represent the standard error of the FOV radius derived from the standard errors of the fit parameters A and k. From the fitted line, the angular FOV was calculated to be 96.6±0.6  deg.

Barrel distortion was evident as expected during endoscopic imaging [Fig. 4(a)]. The distortion constant k and the constant A were determined by fitting Eq. (1) to the data [Fig. 4(b)], $R^{2}=0.9773$ to 0.9927). The values of k and A were then used to determine the FOV radius ($=r_{u}$) based on the radius of the images in pixels ($=r_{d}$). Combining these data for 17 working distances, the angle of the FOV was determined to be 96.6±0.6  deg, which compares favorably to the manufacturer-specified angle of 102 deg [Fig. 4(c)].

### 3.2 Pseudo-RGB Image Reconstruction Enables Region of Interest Placement on Patient Imaging Data

Between January 2019 and June 2021, a total of 13 subjects were recruited to the study. All participants underwent primary surgery for non-secreting pituitary macroadenomas (eight male and five female; age range 27 to 72 years) and provided written informed consent. Of the 13 subjects that underwent surgery, multispectral image data were collected and processed in 12 subjects (Table 1). In one subject, excess bleeding prevented multispectral image acquisition. The broadband halogen light source resulted in an evenly distributed signal across all spectral bands. Acquiring multispectral image data typically lengthened the overall procedure time by <15  min per trial (spectral data captured for a mean of 10.4 min per trial [range 1 to 38 min]).

**Table 1:** Summary of image data collection per patient and comments on analysis for the MSI endoscope.

Case	Pituitary	Adenoma	Blood	Comments
1	3	3	2	—
2	1	2	1	—
3	1	0	1	Not possible to reliably draw tumor ROIs due to bleeding and uncertain of the extent of images
4	0	1	0	Not possible to reliably draw pituitary ROIs as uncertain of the extent of images
5	2	1	1	—
6	0	0	0	Excess bleeding prevented image acquisition
7	0	2	0	—
8	0	2	0	Very small area of pituitary tissue. Signal too low to reliably draw ROI in broadband halogen images
9	0	4	1	Pituitary obscured
10	3	1	1	—
11	2	3	2	—
12	1	1	1	—
13	0	1	1	Pituitary obscured
	13	21	11	45 total regions

**Fig. 5:**
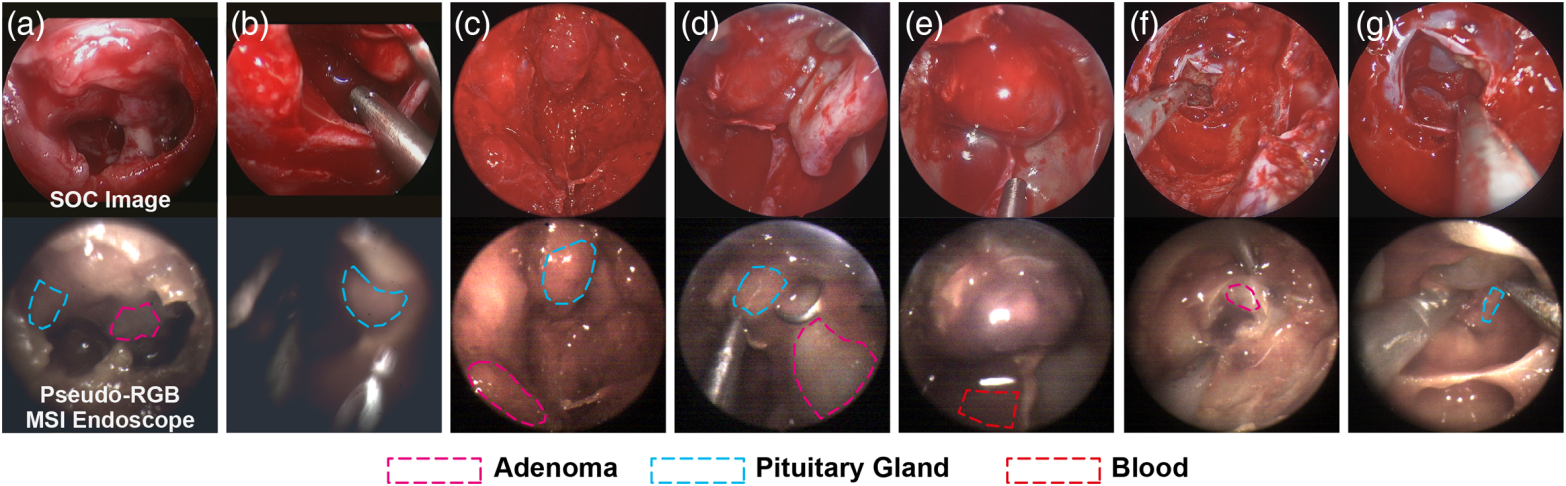
Examples of SOC and pseudo-RGB images from the MSI endoscope taken at the same imaging time point. Regions used for analysis are shown with dotted lines. Images from patients 1, 5, 10, 11, 12, and 13, respectively. Across all ROIs, the average area was 8023 pixels, width 107 pixels, and height 100 pixels.

Images from the standard of care endoscope and pseudo-RGB images from the MSI endoscope were matched at a given imaging time point (see Fig. 2) to define ROIs, guided by the surgical team (authors DB, TS, and JRT; examples shown in Fig. 5). The complex and dynamic surgical field made ROI placement challenging. In some cases, heavy bleeding obscured the surgical field of view [e.g., second image of Fig. 5(a)]. Bleeding must be carefully controlled using continuous well-placed suction and repeated washing of the surgical field of view to obtain usable results. Due to the limited sensitivity of the multispectral camera, longer exposure times were used resulting in low frame rates and motion blur in some images; manual focusing also contributed to image blur [Fig. 5(b)]. Although some cases enabled confident ROI placement in both healthy pituitary and adenoma [Figs. 5(a), 5(c), and 5(d)], others could only identify one tissue type [healthy pituitary Figs. 5(b) and 5(f) and adenoma Fig. 5(e)]. In some patients, the pituitary gland was obscured by other intervening tissues, completely blocking it from visibility (patients 9 and 13) or limiting its visible extent (patients 8 and 12), the latter leading to small ROIs [e.g., Figs. 5(f) and 5(g)]. In some cases, manual manipulation of the surrounding tissue was possible, allowing the pituitary gland to be revealed [Fig. 5(d)], but this was often challenging under guidance from the non-standard and lower-resolution pseudo-RGB images.

Despite these challenges, SOC and MSI images were successfully matched in a total of 45 ROIs that were used for spectral analysis (Table 1). Adenoma ROIs are notably enriched in the dataset because the adenoma was easier to image due to it being ubiquitous, large, in the front and center of the FOV, closer to the endoscope and away from distal blood pooling.

### 3.3 Blood Contributes to Healthy Pituitary Spectra Significantly More Than Adenoma Spectra

**Fig. 6:**
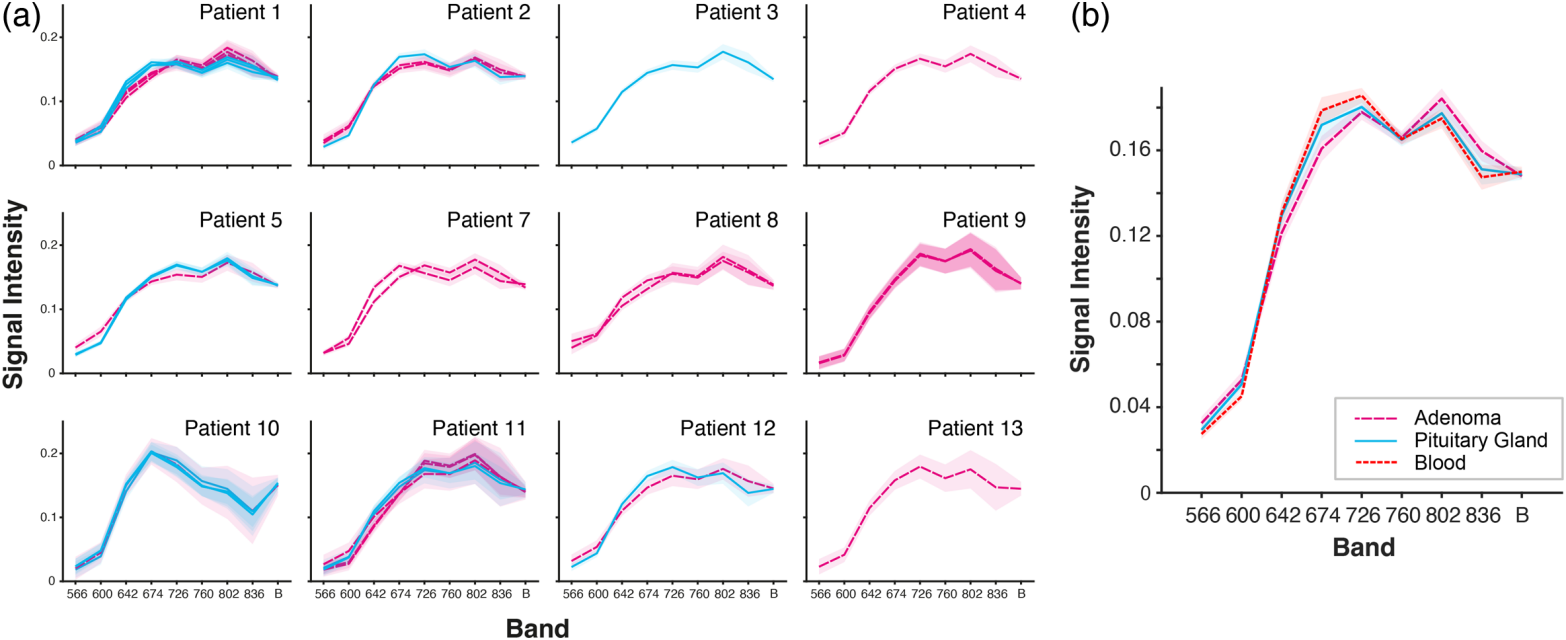
Spectra per-patient and per-pathology class over the whole trial. (a) Average spectra per patient per region to demonstrate the similarity in spectra across the patient cohort and differences according to the different tissue regions. Adenoma is represented by a long dashed pink line; the pituitary gland is represented by a solid blue line. Blood is represented by a short dashed red line. The shaded area represents the standard deviation across pixels within the region of interest. (b) Average spectra per-pathology class. The shaded area represents the standard error across trials.

The mean spectra were extracted from each ROI. The mean spectra per patient per region [Fig. 6(a)] and overall trials [Fig. 6(b)] show the spectra of pituitary tissue, adenoma tissue, and blood being similar, with the spectra of pituitary tissue closer to the blood spectra than the adenoma spectra. These findings confirm that the pituitary gland is well-perfused compared with adenomas, as expected from prior literature.

**Fig. 7:**
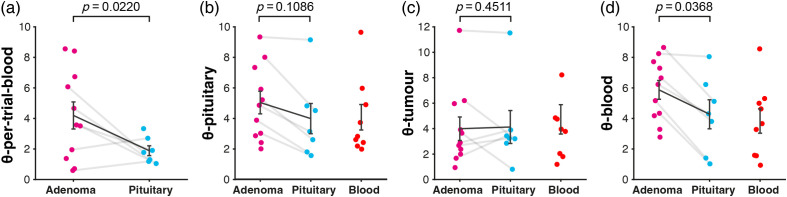
Spectral angle among the mean per-patient per-pathology spectra. (a) Mean per-patient blood spectrum. (b) Mean pituitary spectrum over the whole trial. (c) Mean tumor spectrum over the whole trial. (d) Mean blood spectrum over the whole trial. P values were calculated using the partially paired t test.^30^ n=45 spectra over n=12 patients.

Spectral angles were calculated to compare the mean spectrum from each ROI with four reference spectra (the per-patient mean blood spectrum, the trial-wide mean adenoma spectrum, the trial-wide mean pituitary spectrum, and the trial-wide mean blood spectrum), resulting in four spectral angles per ROI: θ-per-patient-blood, θ-adenoma, θ-pituitary, and θ-blood (Fig. 7). The per-patient blood spectrum was used to give an indication of the relative concentration of blood for adenoma and healthy pituitary. Where a per-patient blood spectrum was not available, the per-patient blood spectrum from an adjacent trial was used.

Spectral angle analysis demonstrates that θ-per-patient blood was significantly smaller for healthy pituitary than for adenoma [p=0.0220, partially paired t test, Fig. 7(a)]. θ-adenoma and θ-pituitary did not show a significant difference [Figs. 7(b) and 7(c)], but θ-blood was significantly smaller for healthy pituitary than for adenoma [p=0.0368, partially paired t test, Fig. 7(d)]. These results suggest a well-perfused pituitary gland relative to adenoma, consistent with previous studies using NBI or ICG to visualize the pituitary gland.

## 4 Discussion and Conclusion

To address the unmet clinical need for better visualization of adenoma in pituitary surgery, an MSI endoscope capable of nine-band multispectral imaging was deployed in a first-in-human pilot study. To achieve real-time acquisition, a multispectral camera based on an SRDA was selected, which provides spectral filtering directly at the sensor pixel in a device that has fewer optical components than traditional spatial- or spectral-scanning multispectral imaging solutions. In addition to real-time acquisition, the use of an SRDA-based camera had several additional advantages: (i) it was easily clipped onto the distal end of the existing CE-marked endoscopic telescope and was vendor agnostic; (ii) no part of the camera head was in direct contact with the patients, so it could be covered with a sterile drape and did not need to resist autoclaving; and (iii) it could be operated in the same way as familiar SOC endoscopes, facilitating a short learning curve for surgeons. These advantages underscore the potential pathway to clinical translation, with negligible additional operator expertise needed and potential for widespread use across laparoscopic imaging applications.

The present study demonstrated that multispectral data can be processed to produce a pseudo-RGB image familiar to the surgeon, albeit at a lower resolution and with a distinct white balance. Nonetheless, a pseudo-RGB image is important for ensuring that patient safety is not compromised during multispectral imaging. The commercial SRDA used had an even distribution of spectral bands from the visible to NIR, thus was not optimized for the detection of any disease-specific spectral signatures or for reconstruction of high-resolution white light images. Due to limitations in the center wavelengths of the spectral bands available on the MSI camera, the pseudo-RGB images were sub-optimal in terms of white balance. Regions were successfully identified between the SOC images and pseudo-RGB images that enabled spectral quantification. Performing spectral angle analysis demonstrated that the absorption of light by blood detected by multispectral imaging can discriminate between an adenoma and a healthy pituitary. These findings are aligned with expectations based on prior experience in NBI but offer a route for direct biomarker quantification in real-time classification and mapping, offering an alternative to the qualitative interpretation process of NBI.

During surgery, the field of view is spatially complex and rapidly changing. Though the prototype MSI endoscope allowed real-time imaging of the surgical field of view, the lower image resolution and frame rate relative to SOC devices meant drawing ROIs for analysis was challenging and required careful human annotations. Further, only simple tissue manipulation was possible when using the multispectral device, meaning the pituitary gland, which must often be revealed by “pushing” occluding tissue out of the way, was challenging. These challenges resulted in a relatively small dataset in the current study.

Blood pooling in the field of view may confound the measured tissue spectra. To minimize this effect, surgeons made every effort to control bleeding using continuous well-placed suction and repeated washing of the surgical field. Despite this, managing blood remained challenging, particularly given the limitations of the current prototype multispectral endoscope. In addition, the pituitary gland was typically hidden by the tumor at the start of surgery and only became visible post-resection, often near areas of distal blood pooling. Although we took care to clear blood from the field of view and to select ROIs that excluded pooled blood, we cannot entirely rule out its contribution to the measured pituitary spectra in this study.

Our first experience of applying an MSI endoscope in pituitary surgery revealed several limitations that will inform future work. First, any MSI endoscope used in future studies should be tailored to provide high-quality white light RGB images as well as MSI. To maximize image resolution, an additional high-resolution RGB sensor could be included in the light path, or improved image processing such as pan-sharpening could be used. Spectral bands could also be targeted for the detection of disease-specific signatures using spectral band optimization,^33^ to reduce the overall number of spectral bands that are sampled. Second, the surgeons had some difficulty using the manual focusing wheel on the multispectral camera head, so incorporating autofocus would be important in future prototypes. Finally, the sensitivity of the system was limited, due to the low quantum efficiency of the underlying sensor (<60%) and the low transmission of the deposited spectral filters (∼40%). A common limitation of SRDA-based cameras, this could be compensated by ensuring the maximum safely permissible illumination power is deposited deliberately over the spectral regions of interest or by incorporating filters with improved optical throughput.

To confirm the findings of the relatively small trial dataset presented here, future trials should aim to include an MSI endoscope with targeted spectra bands, operating at higher sensitivity (and thus frame rates), with autofocusing and real-time preview of the data including RGB displays. With these factors in place, surgeons would be able to better manipulate tissue to reveal tissue types of interest during MSI with real-time feedback for analysis before, during, and after tissue resection. Reaching performance compatible with the existing SOC would mean that MSI devices could be used throughout the surgery, enabling hours of multispectral video to be captured, instead of exchanged at specific time points in the surgical process. A large-scale effort to annotate such videos with tissue labels would be required to build a larger dataset of labeled multispectral images but that could ultimately then be used for tissue-type classification using spectral angle mapping to provide classified images for computer-aided surgical guidance.

In conclusion, MSI endoscopy showed promise for label-free delineation of pituitary adenoma and pituitary tissue based on spectral characteristics. With further development and testing, MSI endoscopy has the potential to improve surgical image guidance, augmenting the surgeon’s vision with label-free functional information to better distinguish tumors from surrounding healthy tissue.

## Data Availability

Code and data related to this paper will be made available on the University of Cambridge data repository after the conclusion of the review process.
